# Supplementary material for: Monoallelic CRMP1 gene variants cause neurodevelopmental disorder
Source: eLife. 2022 Dec 13;11:e80793. doi: 10.7554/eLife.80793 (PMC9803352; doi:10.7554/eLife.80793)
Supplement: Figure 2—source data 1. [file elife-80793-fig2-data1.zip › Fig 2-source data 1/Fig2A-C uncropped and labelled.pptx]

## Slide 1
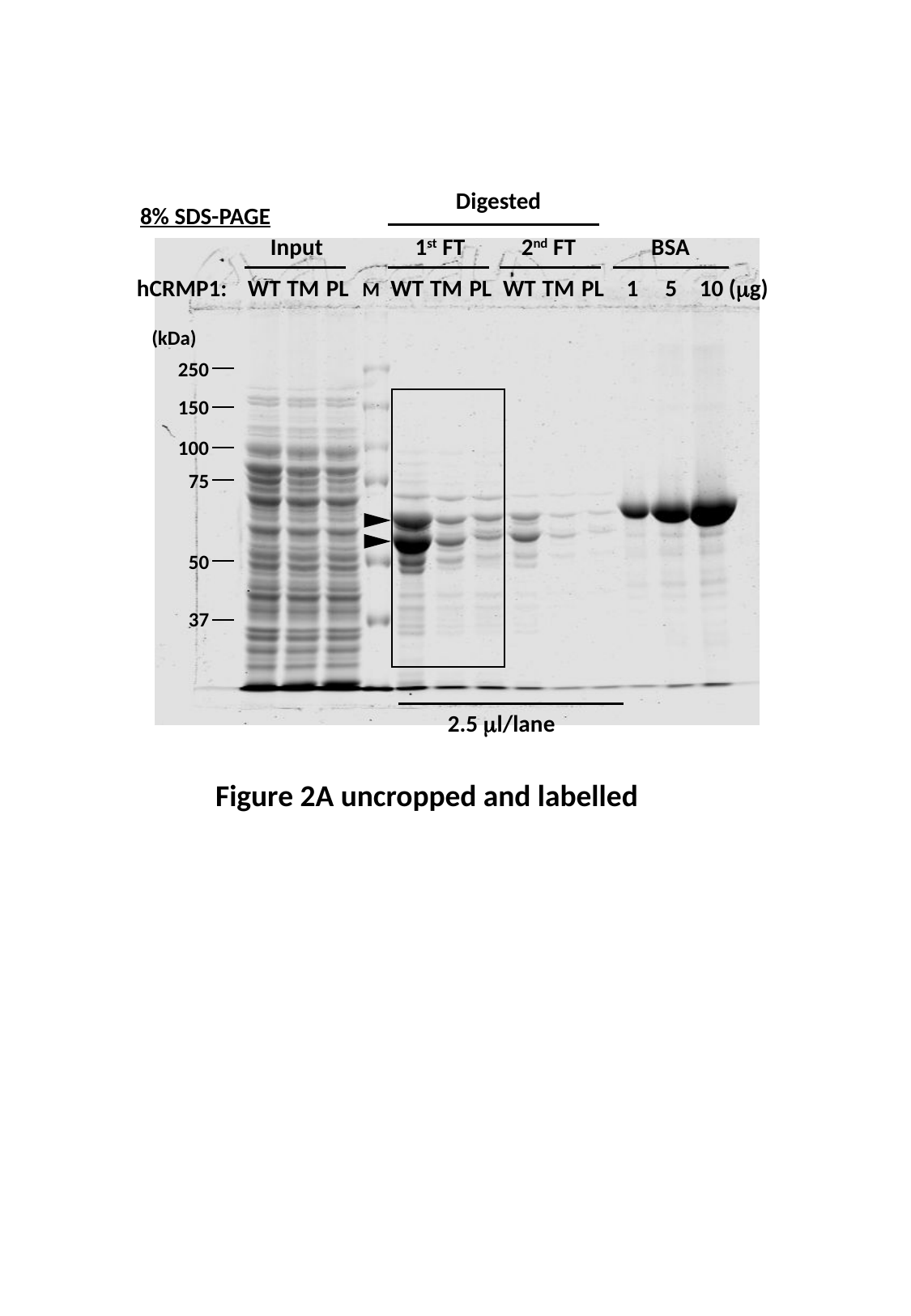

Digested
8% SDS-PAGE
Input
1st FT
2nd FT
BSA
hCRMP1:
WT
TM
PL
WT
TM
PL
WT
TM
PL
1
5
10 (mg)
M
(kDa)
250
150
100
75
50
37
2.5 ml/lane
Figure 2A uncropped and labelled

## Slide 2
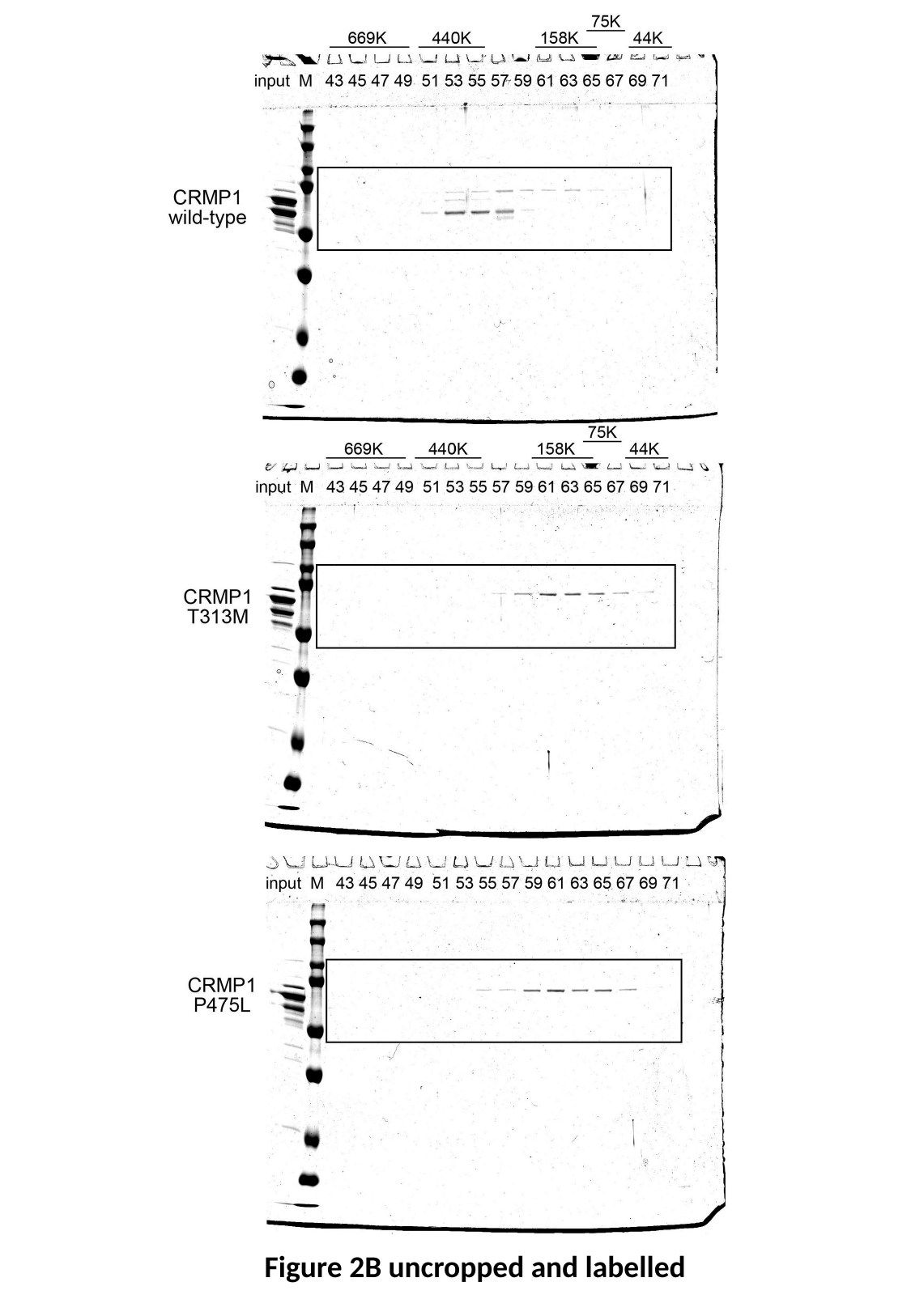

Figure 2B uncropped and labelled

## Slide 3
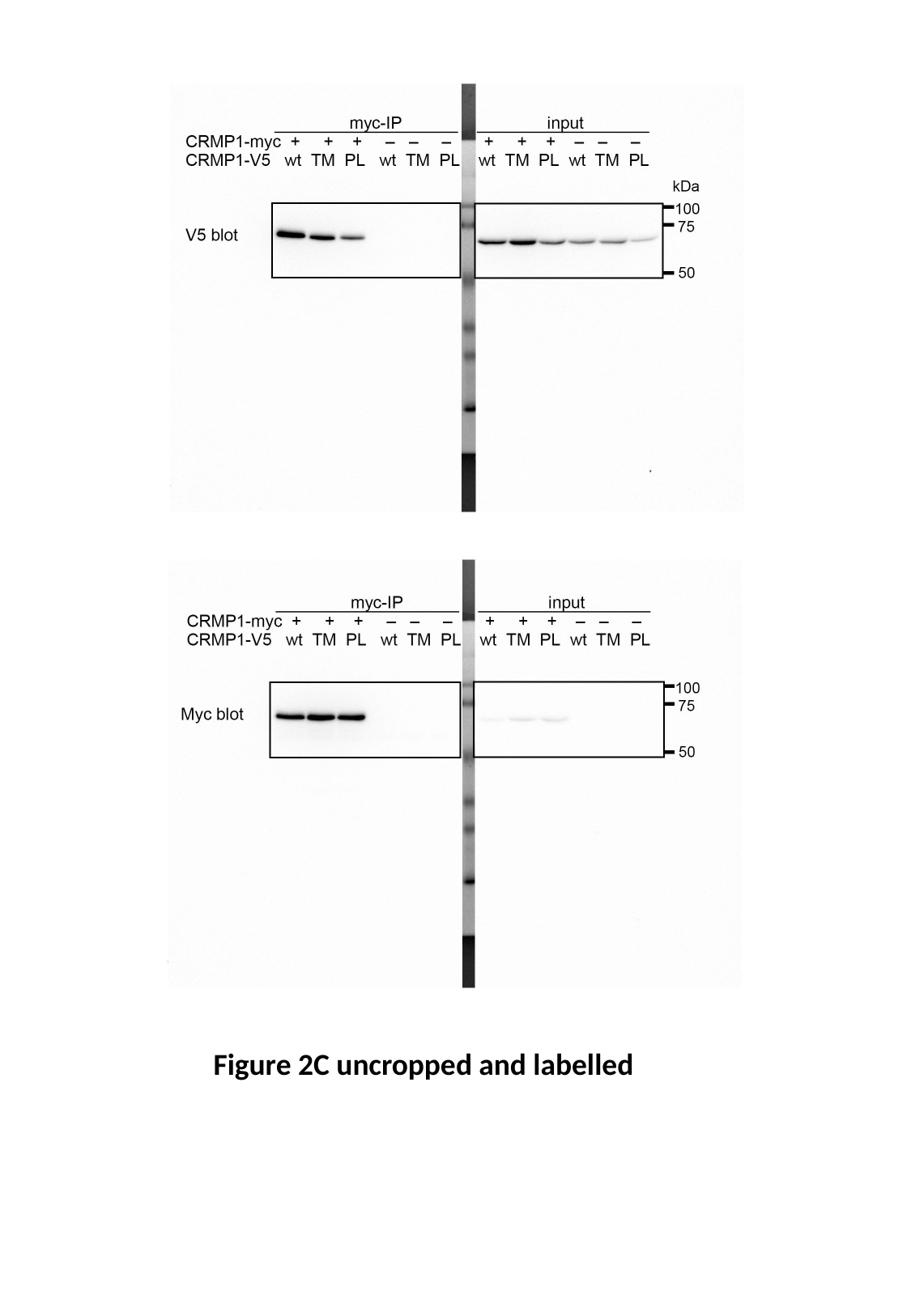

Figure 2C uncropped and labelled
