# Supplementary figures and images for: Monoallelic CRMP1 gene variants cause neurodevelopmental disorder
Source: eLife. 2022 Dec 13;11:e80793. doi: 10.7554/eLife.80793 (PMC9803352; doi:10.7554/eLife.80793)

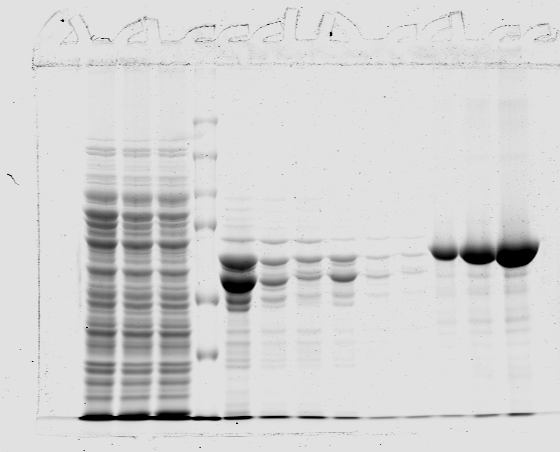

Supplement: Figure 2—source data 1. [file elife-80793-fig2-data1.zip › Fig 2-source data 1/Fig 2A recombinant hCRMP1 CBB original.tif]

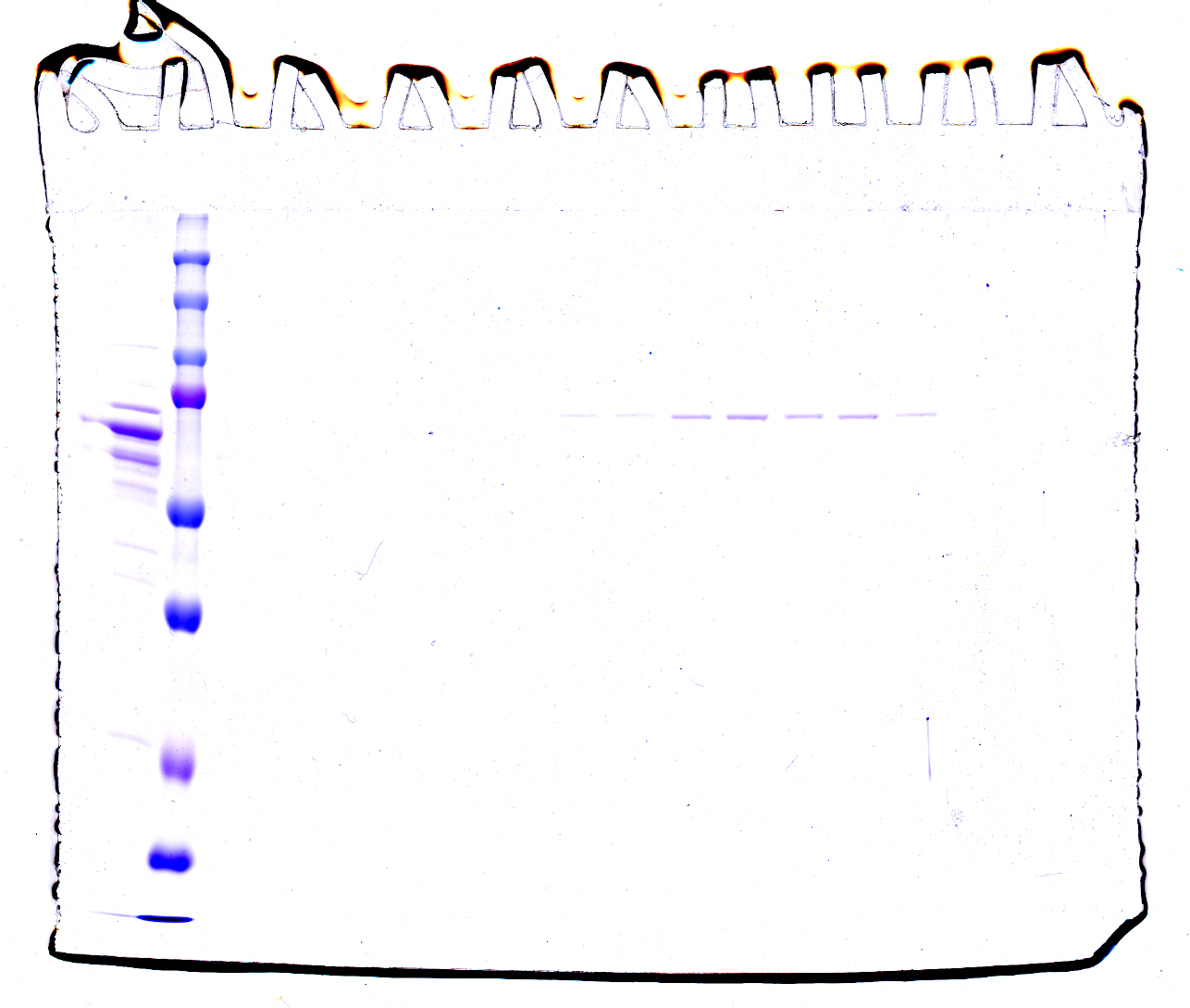

Supplement: Figure 2—source data 1. [file elife-80793-fig2-data1.zip › Fig 2-source data 1/Fig2B PL original.tif]

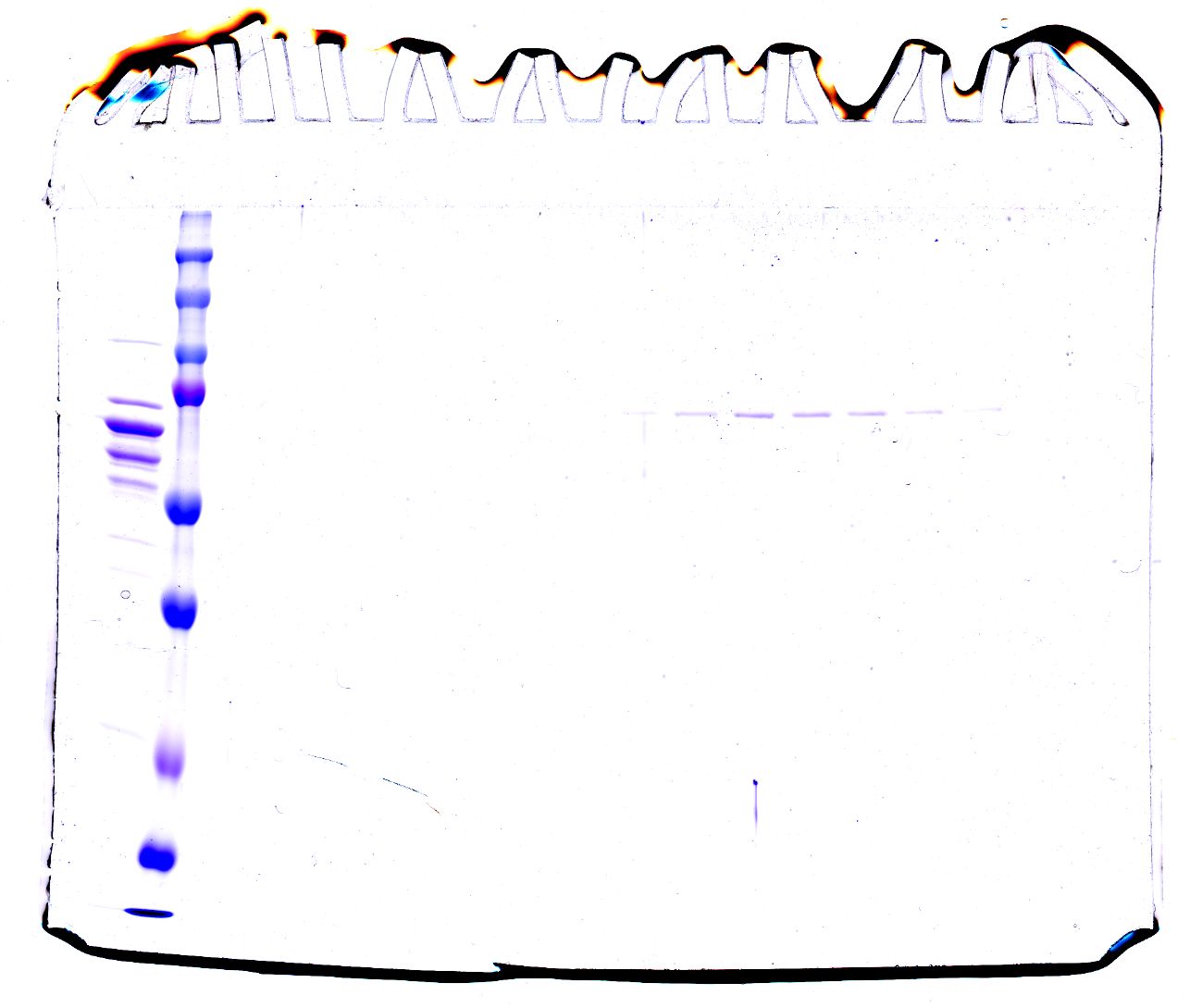

Supplement: Figure 2—source data 1. [file elife-80793-fig2-data1.zip › Fig 2-source data 1/Fig2B TM original.tif]

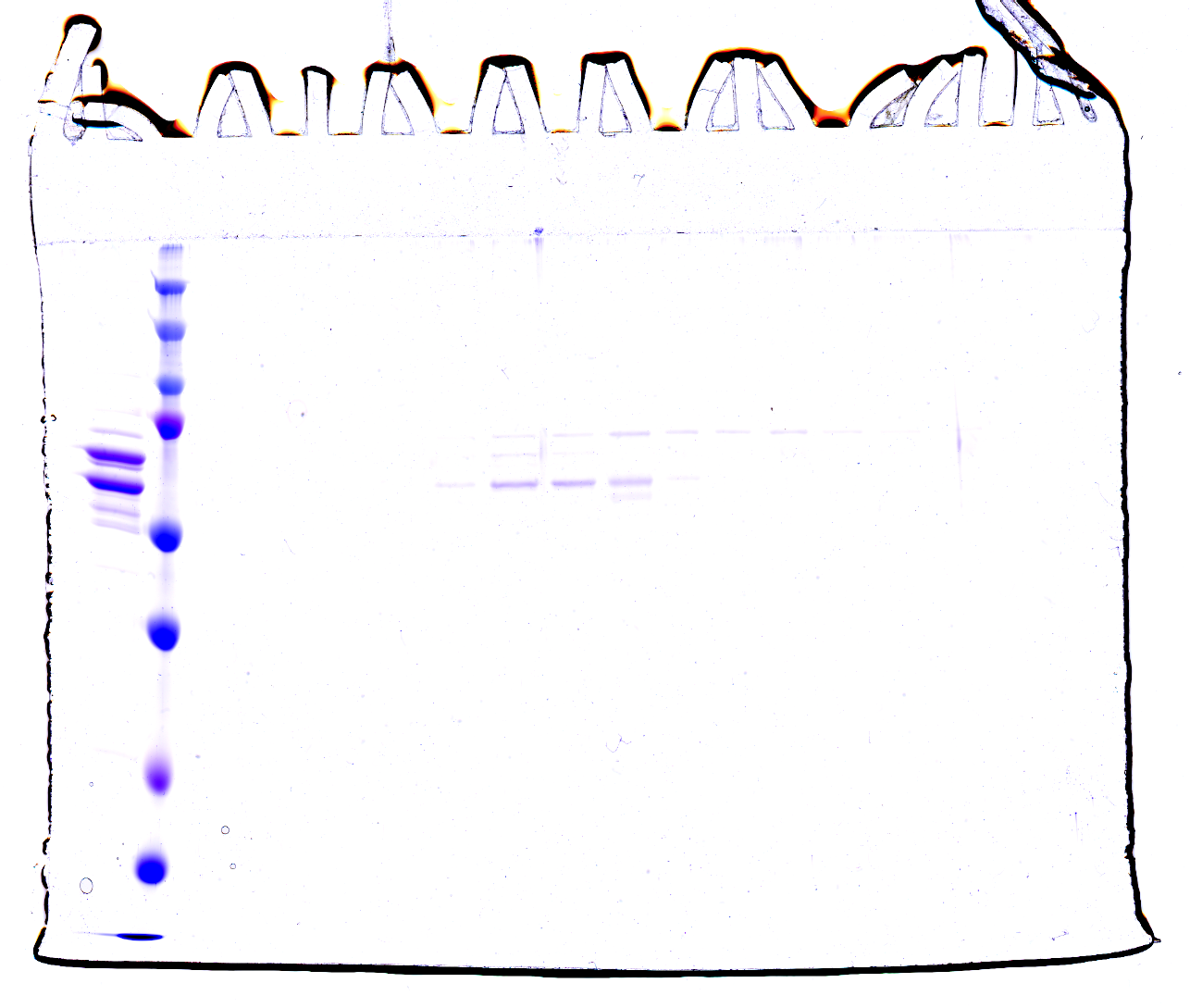

Supplement: Figure 2—source data 1. [file elife-80793-fig2-data1.zip › Fig 2-source data 1/Fig2B wt original.tif]

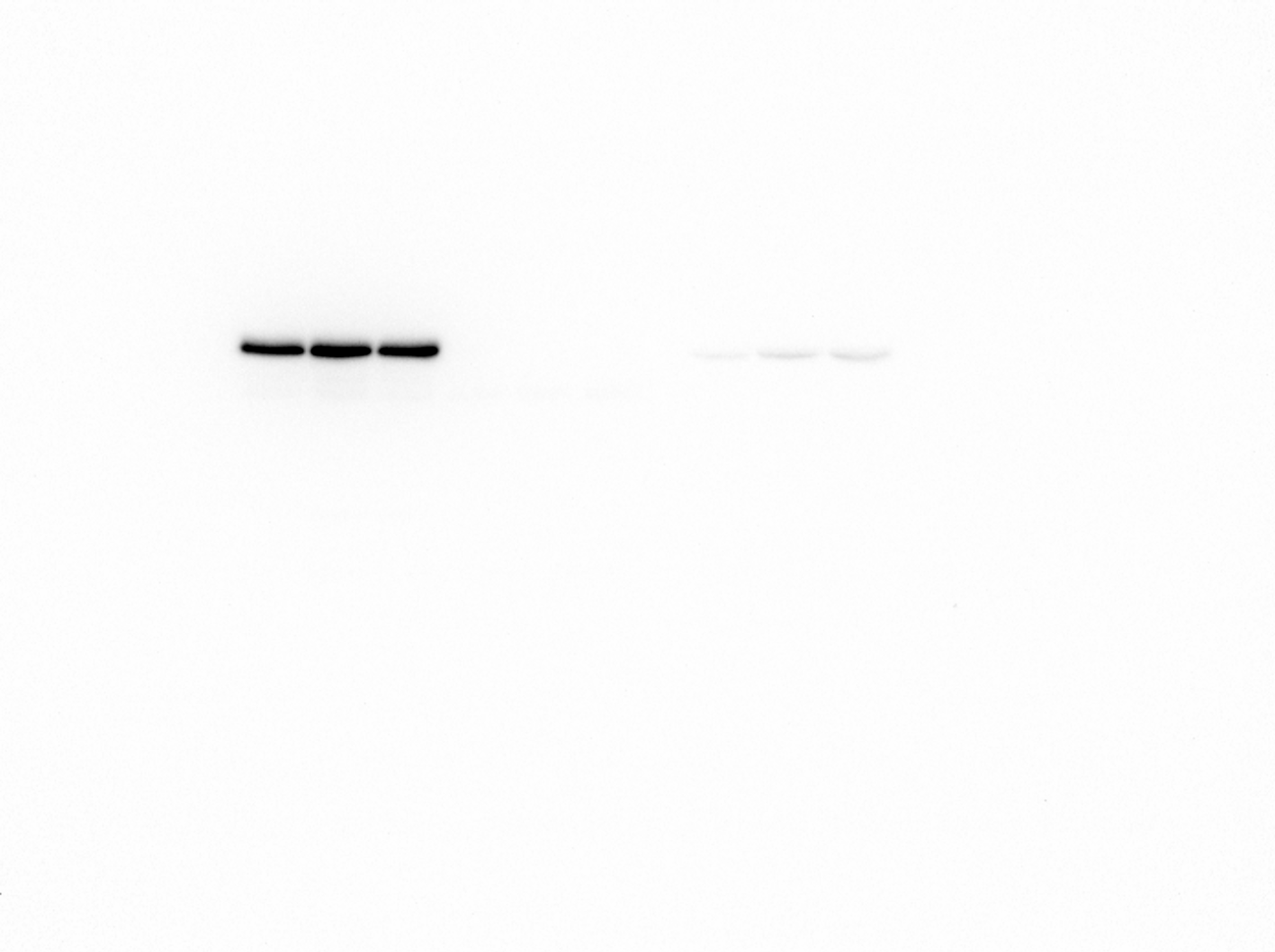

Supplement: Figure 2—source data 1. [file elife-80793-fig2-data1.zip › Fig 2-source data 1/Fig2C Myc 2min.tif]

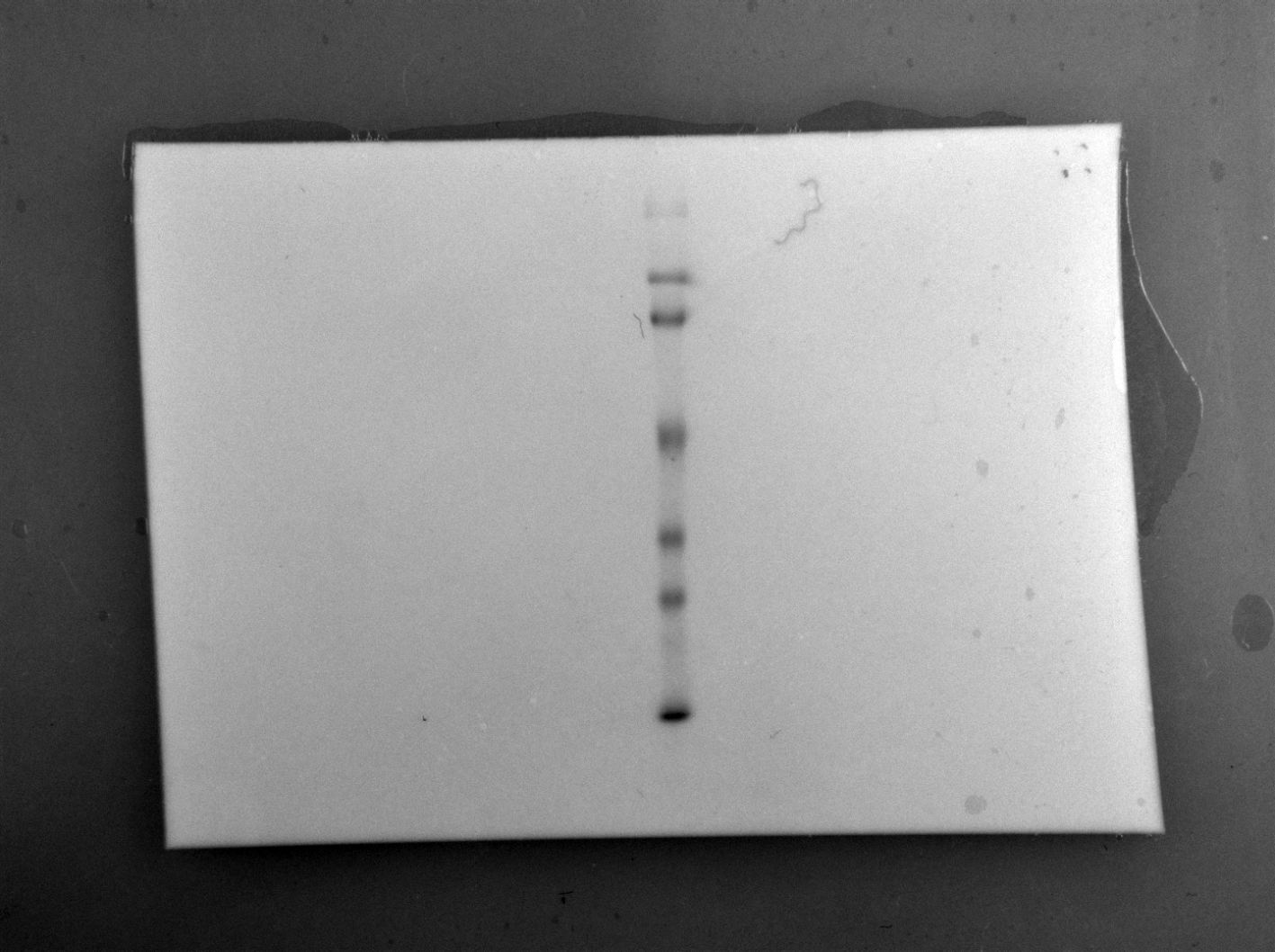

Supplement: Figure 2—source data 1. [file elife-80793-fig2-data1.zip › Fig 2-source data 1/Fig2C Myc marker.tif]

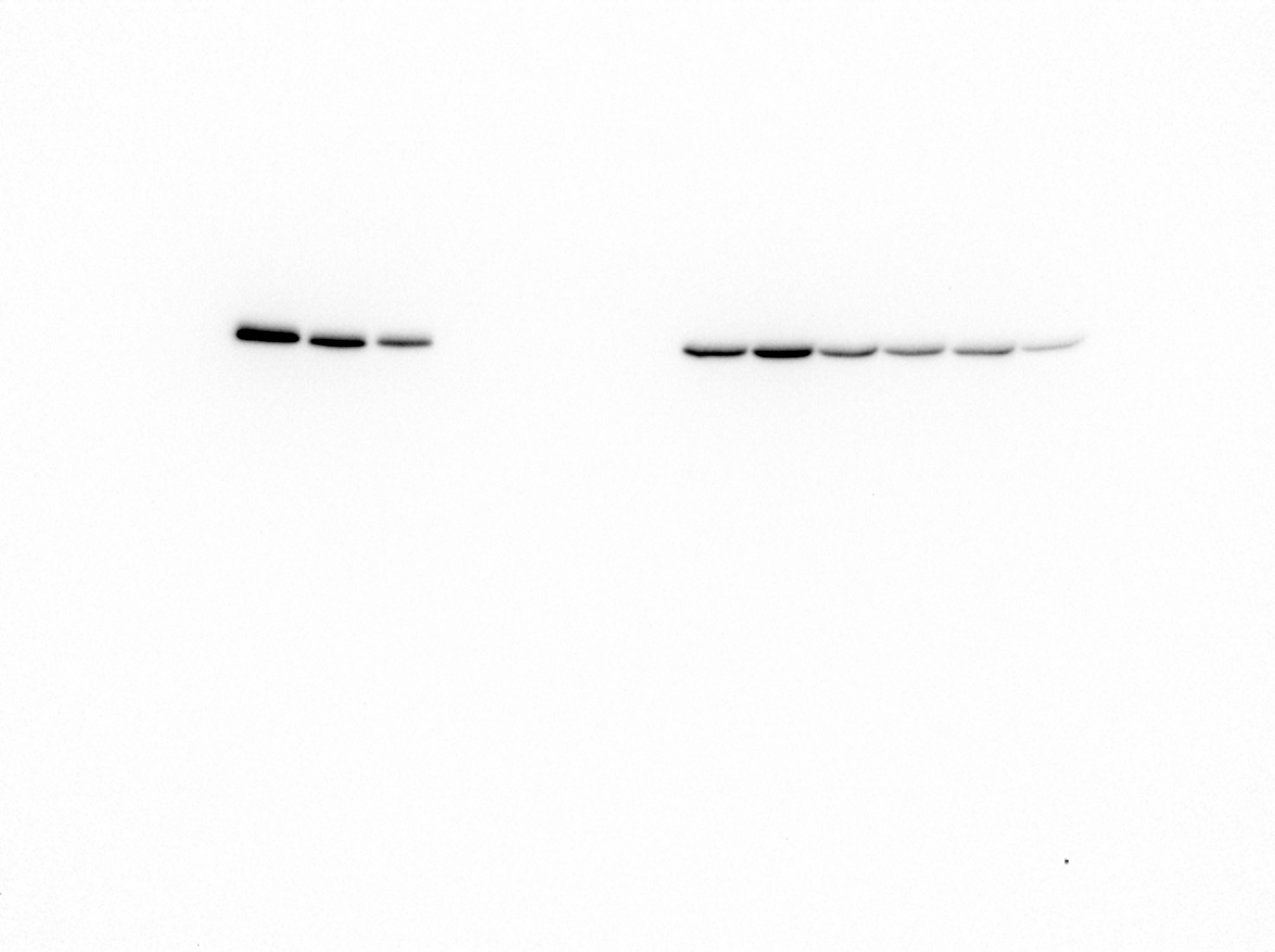

Supplement: Figure 2—source data 1. [file elife-80793-fig2-data1.zip › Fig 2-source data 1/Fig2C V5 60sec.tif]

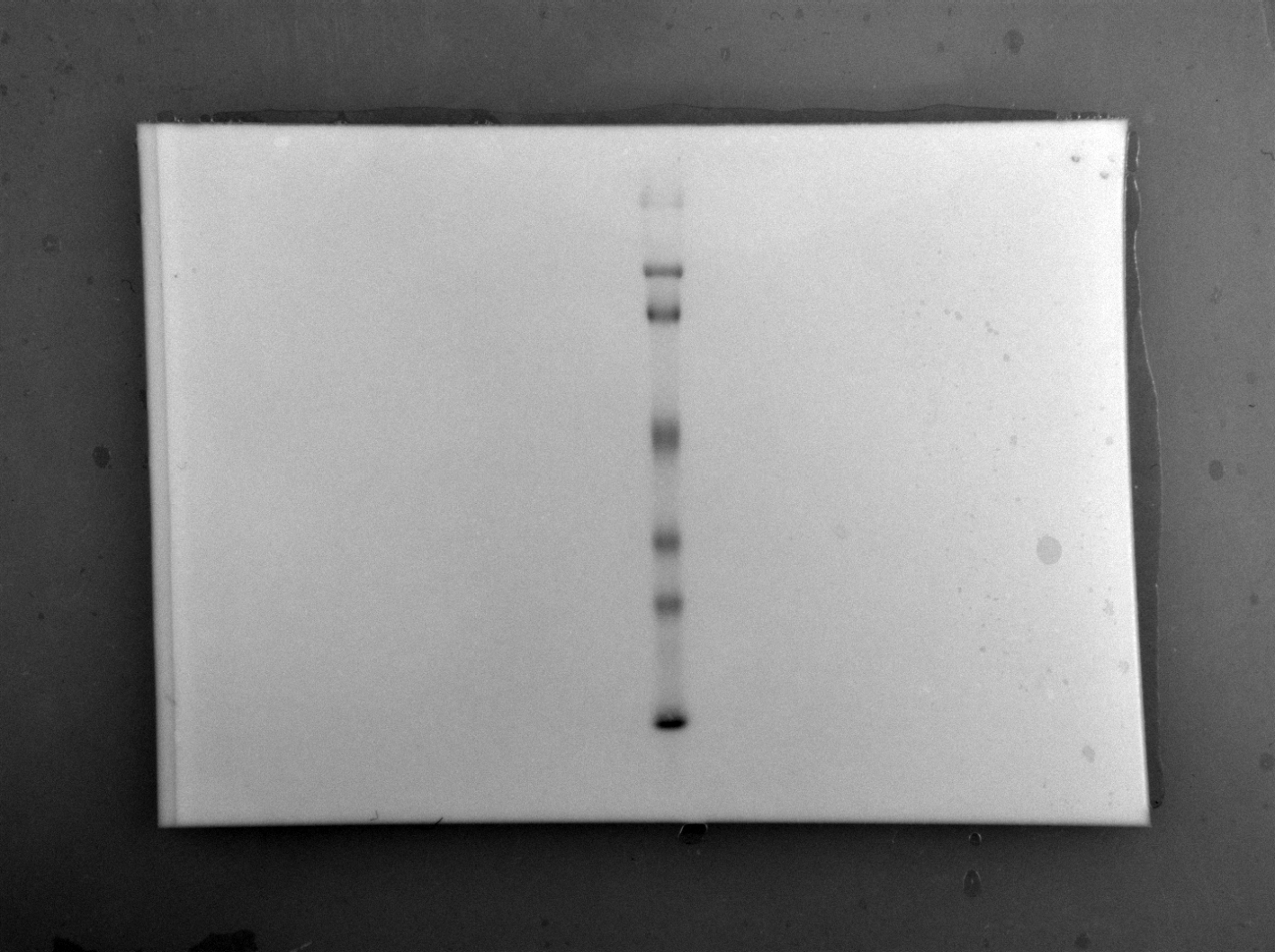

Supplement: Figure 2—source data 1. [file elife-80793-fig2-data1.zip › Fig 2-source data 1/Fig2C V5 marker.tif]

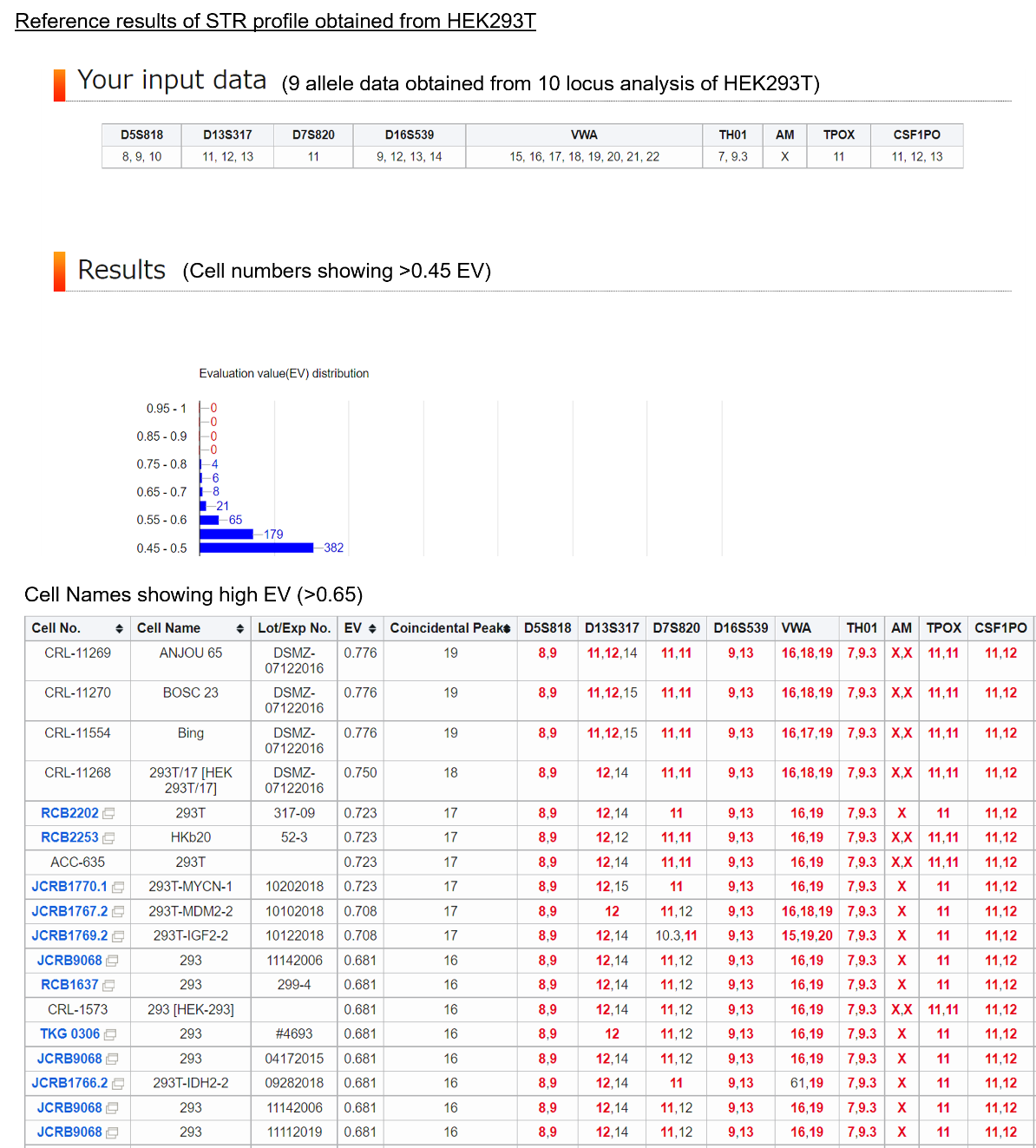


**
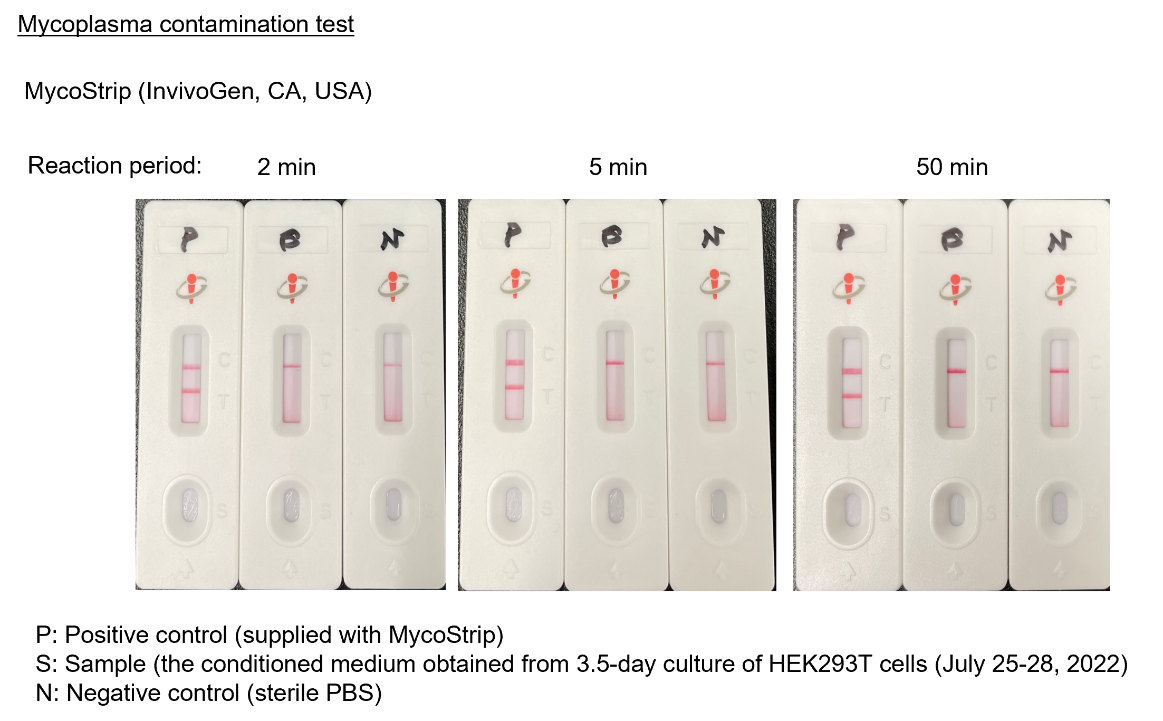
**

Supplement: Supplementary file 1. [file elife-80793-supp1.docx]
